# Editorial: Biomarker Exploration in Neuropsychiatry: Understanding of the Pathophysiology and Therapeutic Implications

**DOI:** 10.3389/fnins.2021.743276

**Published:** 2021-09-29

**Authors:** Hualin Cai, Pei Jiang, Xiangyang Zhang

**Affiliations:** ^1^Department of Pharmacy, Second Xiangya Hospital of Central South University, Changsha, China; ^2^The Institute of Clinical Pharmacy, Central South University, Changsha, China; ^3^Institute of Clinical Pharmacology, Jining First People's Hospital, Jining Medical University, Jining, China; ^4^CAS Key Laboratory of Mental Health, Institute of Psychology, Chinese Academy of Sciences, Bejing, China; ^5^Department of Psychology, University of Chinese Academy of Sciences, Bejing, China

**Keywords:** genomics, proteomics, metabolomics, neuroimaging, biomarkers, psychiatry

This Research Topic, consisting of fifteen research and review articles, aimed to expand our knowledge and understanding of inspiring advances in various technologies which have provided us with new avenues to investigate biomarkers in neuropsychiatric disorders. Among these technologies, rapid developments in bioinformatics allow us to use computation to extract key information from biological databases. It includes the collection, manipulation and modeling of data for analysis, visualization, or prediction through the development of algorithms and software. It gives the researchers the new skills of finding the needle in a haystack. Here, Huang et al. searched the Gene Expression Omnibus (GEO) database for microarray studies of fibroblasts, lymphoblasts, and post-mortem brains of schizophrenia patients. They demonstrated that high FOS expression in non-neural peripheral samples and low FOS expression in brain tissues exist in schizophrenia patients. Since FOS is thought to play an important role in the pathophysiology of schizophrenia (Monfil et al., 2018), they further carried out Kyoto Encyclopedia of Genes and Genomes (KEGG) enrichment analysis and revealed that “amphetamine addiction” was among the top 10 significantly enriched KEGG pathways. Likewise, the protein-protein interaction network analysis indicated that proteins closely interacting with FOS-encoded protein were also involved in the amphetamine addiction pathway. To the end, the authors propose that FOS and amphetamine-related genes may be involved in schizophrenia pathogenesis and represent novel biomarkers for its diagnosis in clinical practice.

In addition to bioinformatics, the analysis of structural covariance networks (SCNs) has been successfully applied to obtain the abnormality in brain connectivity of neuropsychiatric disorders. This technology based on voxel-based morphometry, can generate a map of correlation between the gray matter volume of a targeted brain region and the other regions (Zielinski et al., 2010). As such, SCNs analysis is regarded as a potential tool to reflect developmental coordination or synchronized maturation between different regions of the brain. Here, Wang et al. investigated abnormalities in SCNs between hippocampal subfields and the whole cerebral cortex in amnestic mild cognitive impairment (aMCI) and in subcortical vascular mild cognitive impairment (svMCI) patients, and then made comparisons of these abnormalities between the two subtypes. They found: (1) significant correlations between hippocampal subfields, fusiform gyrus, and entorhinal cortex in gray matter volume in each group; (2) as compared to normal controls, an increased association between the left CA1/CA4/DG/subiculum and the left temporal pole in aMCI group, and covariations between the hippocampal subfields (bilateral CA1, left CA2/3) and the orbitofrontal cortex in svMCI group; (3) an decreased association between hippocampal subfields and the right fusiform gyrus, as well as increased association between the bilateral subiculum/presubiculum and bilateral entorhinal cortex, when aMCI group was compared to svMCI group. These findings demonstrate that there is altered whole-brain structural covariance of the hippocampal subfields in svMCI and aMCI patients and provide insights to the imaging biomarkers of different mild cognitive impairment subtypes.

Omics-technologies, including genomics, transcriptomics, proteomics, and metabolomics, have revolutionized biomedical research over the past two decades, and are now poised to play a transformative role in the explorations of biomarkers for neuropsychiatric disorders. In the study by Brandão-Teles et al. using human oligodendrocyte cell line as the model, the authors performed a mass spectrometry-based, bottom-up shotgun proteomic analysis to identify different effects triggered by typical (chlorpromazine and haloperidol) and atypical (quetiapine and risperidone) antipsychotic drugs. The results showed that the two types of antipsychotic drugs shared common effects on spliceosome machinery, eukaryotic initiation factor-2 signaling, rapamycin (mTOR) signaling pathway, ubiquitination pathway, energy metabolism, and 14-3-3 family proteins. Drug-specific differences triggered by antipsychotics were also observed, suggesting that (1) risperidone has more pathways in common with the two typical antipsychotics than with quetiapine; (2) chlorpromazine appears to increase protein levels, whereas haloperidol has the opposite action; (3) quetiapine alters fewer proteins than the other three drugs. Although *in vitro* studies may have discrepancies with the *in vivo* scenario, these detailed findings shed light on the biochemical pathways underlying the mechanisms these antipsychotics, which may guide the identification of novel treatment biomarkers and the development of new therapeutic strategies. To decipher the complexity and heterogeneity of neuropsychiatric disorders, integration of proteomics, bioinformatics and systems biology can be even more powerful (Guingab-Cagmat et al., 2013). Hypothalamus has important modulatory functions in the brain and controls the activity of hypothalamic-pituitary-adrenal (HPA) axis responding to stress (Myers et al., 2014). Herein, Zhang et al. established a chronic corticosterone-induced mouse model of depression to assess the antidepressant-like effects and mechanisms of baicalin, using a combined method of proteomics and systems biology. Using proteomics, they found 370 differentially expressed proteins in the depression rat model after baicalin treatment, including 114 up-regulation and 256 down-regulation in hypothalamus. Then systems biology analysis was performed to narrow down the information, and indicates that differentially expressed proteins are focused on phosphoserine binding and phosphorylation, especially participate in glucocorticoid receptor (GR) signaling pathway. Finally, the findings demonstrate that baicalin can reduce hypothalamic GR phosphorylation to remodel and normalize the negative feedback of HPA axis.

Metabolomics is a global approach to understanding regulation of metabolic pathways and networks of a biological system, and emerges as another powerful tool for identification of biomarkers in central nervous system research. Here, for the first time, Papadopoulou et al. conducted a targeted metabolomics study based on mass spectrometry platform, to compare the serum metabolomes including up to 300 metabolites of a characterized group of mothers suffering from post-partum depression (PPD) and a control group of mothers. They demonstrated increased levels of glutathione-disulfide, adenylosuccinate, and adenosine triphosphate (ATP) in the PPD group, which are involved in oxidative stress, nucleotide biosynthesis and energy production pathways. Moreover, the metabolomic findings are further validated in a separate cohort of PPD mothers and controls. Importantly, the data indicate that PPD-induced molecular alterations are detectable in the periphery and may open up new perspectives for more studies aiming at early detection and diagnosis for PPD. Although studies in peripheral samples are essential for developing safe, non-invasive diagnostic and screening approaches for neuropsychiatric disorders, the specificity of disease-related molecular correlates in peripheral material has always been one of the biggest challenges we are facing (Filiou and Turck, 2011). Herein, Zhou et al. compared plasma free fatty acid (FFA) levels and profiles among healthy controls (HCs), affective psychosis (AP) patients with bipolar disorder/major depression as disease-controls, and first-episode antipsychotic-naïve schizophrenia (FEANS) patients, using a more focused quantitative metabolomic strategy performed with capillary gas chromatography. The FFAs are involved in many important biochemical reactions such as membrane regeneration, oxidation, and prostaglandin production, which have important implications in schizophrenia pathology (Yao and Reddy, 2002). Interestingly, as compared with HCs, a significant reduction of total FFAs levels and disrupted metabolism of fatty acids especially in saturated and n-6 fatty acid families were observed in FEANS patients. In the meantime, the reduction of 16:0, 18:2n6c, and 20:4n6 levels were merely detected in FEANS patients rather than in AP patients, which have extensive symptomatic and genetic overlap with schizophrenia. Collectively, these findings could help us better understand the lipid metabolism with regard to schizophrenia pathophysiology and provide insights into searching for disease-specific biomarkers.

Despite the abovementioned “omics” technologies, discovery of biomarkers is sometimes based on an underlying assumption regarding the pathogenesis and pathophysiology of the disease, a traditional strategy which can be termed as “hypothesis-driven.” Several hypothesis-driven biomarker verification studies have been collected in this Research Topic. As it is well-known that altered HPA axis function has played an important role in the neurodegenerative process in schizophrenia (Walker and Diforio, 1997), here Liu et al. performed a quantitative analysis of the peripheral blood mRNA expression of GR, GR transcripts containing exons 1B (GR-1B), and neuron specific enolase (NSE) genes and serum cortisol and NSE (a specific serum marker for neuronal damage). In this study, they found abnormal serum levels of cortisol in chronic schizophrenia patients and NSE protein levels in first-episode schizophrenia patients, respectively. Additionally, further evidence of altered NSE mRNA in chronic schizophrenia, GR mRNA in both first-episode and chronic schizophrenia and decreased GR-1B mRNA in chronic vs. first-episode schizophrenia were identified. These abnormalities particularly implicate the disrupted HPA axis and the dysregulation of GR mRNA and protein expression can be manifested at different stages of schizophrenia. Based on the assumption that abnormal immune system and immunological responses may be related with the etiology of schizophrenia (Monji et al., 2013), here another investigation implemented by Zhu et al. recruited 69 FEANS patients, 87 chronic schizophrenia patients and 61 HCs. They demonstrated that serum TNF-α and IL-1β levels in chronic schizophrenia were significantly higher than HCs, whereas their concentrations in FEANS patients were significantly lower as compared with both chronic schizophrenia patients and HCs. A moderate correlation between serum TNF-α/IL-1β levels and PANSS negative subscale was also found in chronic schizophrenia patients instead of FEANS. They concluded that altered immune response in chronic patients may be associated with the progression, psychotropic drugs, or other factors occur during chronic stage. The ATP-binding cassette subfamily B member 1 (ABCB1) gene is a multidrug resistance protein 1 (MDR1) gene encodes p-glycoprotein (P-gp). This protein expressed in the blood-brain barrier protects the brain from drugs or neurotoxic substances, and thus may play an important role in the bioavailability and response to central nervous system drugs (de Klerk et al., 2013). Here, Shan et al. explored the potential correlations of therapeutic responses with selective serotonin reuptake inhibitors (SSRIs) and serotonin-norepinephrine reuptake inhibitors (SNRIs) in a local Chinese Han population consisting of 292 patients with major depression. Interestingly, they found that the ABCB1 gene polymorphisms (the TT genotype of rs2032583) could be a predictive factor of better treatment responses to SNRIs in the Chinese population, which however, ought to be replicated in future studies with a larger cohort. Timely updates of underlying mechanisms and pathophysiology can help to generate new hypotheses, and thus may greatly accelerate new drug development and biomarker discovery. Here, Gong et al., for the first time, provided new evidence highlighting the involvement of biomarkers of renin-angiotensin system (RAS) in inflammation-impeded brain insulin pathway, which results in compromised neurobehavioral changes. The data suggests that inhibition of RAS seems to be a promising strategy to block or even cut-off the cross-talk and vicious cycle between RAS and immune system, which could serve as a potential therapeutic target for the inflammation-associated neuropsychiatric disorders such as depression.

Second-generation antipsychotics, also named atypical antipsychotic drugs (AAPDs), are first-line antipsychotics with greater improvement of negative symptoms and fewer extrapyramidal symptoms than first-generation antipsychotic drugs. However, metabolic side effects, especially well-documented dyslipidemia, type II diabetes, and weight gain induced by prolonged usage of AAPDs raise the risk of cardiovascular diseases, resulting in patient non-compliance, relapse, and increased mortality (Mitchell et al., 2013). Since related side effects could vary from patient to patient, potential biomarkers screening to predict the risk of metabolic side effects of antipsychotic drug is becoming a hot area in pharmacogenomic research. Here, Li N. et al. systematically reviewed recently updated findings on the pharmacogenomics and gene polymorphisms related to lipid disturbances of AAPDs, with the aim to find the possible relations between central and peripheral pathways. In the review, evidence indicated that in central nervous system, HTR2C, DRD2, LEP, NPY, MC4R, BDNF, CNR1 polymorphisms play an important role in regulating food intake, and they can be affected by AAPDs. Meanwhile, the lipid metabolism in peripheral tissues may be altered by the SNPs of LEP, NPY, MC4R, CNR1, INSIG2, and ADRA2A. Among these genes, complex pathways are involved in the modulation of energy intake and energy expenditure, representing orexigenic and anorexigenic mechanisms. The disturbance in glucose metabolism is another aspect of AAPD-induced adverse effects. Recently, accumulating evidence suggests that Wnt signaling pathway has a pivotal role in the pathogenesis of schizophrenia and molecular cascades of antipsychotic actions. TCF7L2, the key effector of Wnt signaling pathway, is strongly associated with glucose homeostasis (Singh, 2013). Here, a mechanism study performed by Li R. et al. explore the characteristics of metabolic disturbance induced by olanzapine and their associations to TCF7L2. Significantly increased body weight, fasting insulin, homeostasis model assessment-insulin resistance index, and TCF7L2 protein expression in liver, skeletal muscle, and adipose tissues were found, which could be reversed by metformin co-treatment. The results illustrate that TCF7L2 overexpression in liver, skeletal muscle, and adipose tissues may serve as a potential mechanism/biomarker indicative of olanzapine induced metabolic changes. Anti-N-methyl-D-aspartate receptor (anti-NMDAR) encephalitis is an autoimmune encephalitis and is often combined with psychiatric symptoms, such as severe hallucination, delusion, and aggressive behaviors (Warren et al., 2018). Some scholars hypothesized that NMDAR dysfunction was the “final common pathway” underlying the pathogenesis of schizophrenia (Wang et al., 2017). Therefore, it is believed that schizophrenia and anti-NMDAR encephalitis may have certain shared underpinnings and could be on the same spectrum (Maneta and Garcia, 2014). Here, Yang et al. reported a series of three cases of anti-NMDAR encephalitis with psychiatric symptoms. Using the anti-NMDAR antibodies in cerebrospinal fluid and serum as the treatment biomarker, they found that the AAPD clozapine may be effective, if the psychiatric symptoms could not be controlled after intravenous immunoglobulin and hormone therapy. It is noteworthy that quetiapine and aripiprazole were ineffective and olanzapine even worsened the psychiatric symptoms of these cases. It is speculated that the distinct affinity of receptor subtypes may play a role but the exact mechanism warrants further investigations.

Previously, metabolomes illustrating what happened in the organism, were generally considered as end products of genomes and thus most of their fate was to be excreted as waste. However, new advances have modified the theory and demonstrated that downstream metabolite concentrations can regulate upstream gene expression, which can in turn exert certain therapeutic effects by regulating cascades of biological process and metabolic activity (Bradley et al., 2009). Hydrogen sulfide (H_2_S) is regarded as the one of the most important endogenous gasotransmitter, and plays a number of roles in the central nervous system under pathological and physiological conditions such as anti-inflammation, cytoprotection, anti-apoptosis, and antioxidation (Kimura, 2013). Endogenous H_2_S is mainly produced from L-cysteine, cystathionine, and β-mercaptopyruvic acid in mitochondria. Here, Wen et al. carried out a study showing the multifaceted vasoprotection of H_2_S on cerebral ischemia/reperfusion injury. They demonstrated that both endogenous and exogenous H_2_S had prominent protection on vasomotor dysfunction and neuronal injury induced by transient middle cerebral artery occlusion in rats. Specifically, K_Ca_ channel might be involved in the cerebrovascular relaxation to H_2_S, which is endothelium-dependent. Increasing evidence suggests that many neurosteroids have neuroprotective properties on the central nervous system. Although the mechanisms are far from fully elucidated, progesterone (PROG) and its active metabolite allopregnanolone (ALLO) have eminent neuroprotective effects against some nervous system diseases, including traumatic brain injury and spinal cord injury and schizophrenia-related cognitive impairment (Cai et al., 2018). Herein, Cao et al. investigated the capability of different dose of PROG/ALLO (8.16 mg kg^−1^) on ameliorating ketamine-induced cognitive deficits, and related mechanisms via the progesterone receptor membrane component 1 (PGRMC1) pathway in hippocampus and prefrontal cortex were elaborated simultaneously. The results showed that PROG or ALLO could reverse the impaired spatial learning and memory abilities induced by ketamine, which was accompanied with the upregulation of PGRMC1/EGFR/GLP-1R/PI3K/Akt pathway in the two brain regions. Additionally, the coadministration of PGRMC1 specific inhibitor AG205 abolished their neuroprotective effects, suggesting the crucial role of PGRMC1 underlying the neuroprotection mechanisms of PROG/ALLO. The study may shed light on future clinical practice utilizing neurosteroid adjunctive therapy to enhance cognitive function in neuropsychiatric diseases by targeting on PGRMC1 signaling.

In summary, this collection highlights the wide range of technological strategies for discovery and validation of biomarkers in neuropsychiatric disorders. And new understanding of the mechanisms underlying the therapeutic effects of some metabolic biomarkers are also discussed. These studies enrich the pool of potential biomarkers for future clinical application, which not only facilitate objective diagnosis of neuropsychiatric diseases but also help develop promising avenues to improve treatment responses.

## Author Contributions

HC wrote the draft. PJ and XZ provided comments for revisions. All authors approved the publication of this editorial.

## Funding

This work was supported in part by the grants from Hunan Provincial Natural Science Foundation of China [2021JJ30922], Hunan Provincial Health Commission Research Project [202113010595], Wu Jieping Medical Foundation Funded Special Clinical Research Project [320.6750.2020-04-2], Changsha Municipal Natural Science Foundation [kq2007045] and the Fundamental Research Funds for the Central Universities of Central South University [2019zzts1049, 2020zzts884, 2021zzts1073].

## Conflict of Interest

The authors declare that the research was conducted in the absence of any commercial or financial relationships that could be construed as a potential conflict of interest.

## Publisher's Note

All claims expressed in this article are solely those of the authors and do not necessarily represent those of their affiliated organizations, or those of the publisher, the editors and the reviewers. Any product that may be evaluated in this article, or claim that may be made by its manufacturer, is not guaranteed or endorsed by the publisher.
